# Characterization of the yeast ionome: a genome-wide analysis of nutrient mineral and trace element homeostasis in *Saccharomyces cerevisiae*

**DOI:** 10.1186/gb-2005-6-9-r77

**Published:** 2005-08-30

**Authors:** David J Eide, Suzanne Clark, T Murlidharan Nair, Mathias Gehl, Michael Gribskov, Mary Lou Guerinot, Jeffrey F Harper

**Affiliations:** 1 Department of Nutritional Sciences, University of Wisconsin-Madison, Madison, WI 53706, USA; 2 San Diego Supercomputer Center, University of California-San Diego, La Jolla, CA 92903, USA; 3 Biochemistry Department, University of Nevada, Reno, Nevada 89557, USA; 4 Department of Biological Sciences, Dartmouth College, Hanover, NH 03755, USA

## Abstract

**Background:**

Nutrient minerals are essential yet potentially toxic, and homeostatic mechanisms are required to regulate their intracellular levels. We describe here a genome-wide screen for genes involved in the homeostasis of minerals in *Saccharomyces cerevisiae*. Using inductively coupled plasma-atomic emission spectroscopy (ICP-AES), we assayed 4,385 mutant strains for the accumulation of 13 elements (calcium, cobalt, copper, iron, potassium, magnesium, manganese, nickel, phosphorus, selenium, sodium, sulfur, and zinc). We refer to the resulting accumulation profile as the yeast 'ionome'.

**Results:**

We identified 212 strains that showed altered ionome profiles when grown on a rich growth medium. Surprisingly few of these mutants (four strains) were affected for only one element. Rather, levels of multiple elements were altered in most mutants. It was also remarkable that only six genes previously shown to be involved in the uptake and utilization of minerals were identified here, indicating that homeostasis is robust under these replete conditions. Many mutants identified affected either mitochondrial or vacuolar function and these groups showed similar effects on the accumulation of many different elements. In addition, intriguing positive and negative correlations among different elements were observed. Finally, ionome profile data allowed us to correctly predict a function for a previously uncharacterized gene, *YDR065W*. We show that this gene is required for vacuolar acidification.

**Conclusion:**

Our results indicate the power of ionomics to identify new aspects of mineral homeostasis and how these data can be used to develop hypotheses regarding the functions of previously uncharacterized genes.

## Abstract 2

The accumulation of thirteen minerals was assayed in 4,385 yeast mutant strains, identifying 212 strains that showed altered ionome (mineral accumulation) profiles.

## Background

Living cells are composed of a large variety of chemical elements. In addition to carbon, nitrogen, and oxygen, cells require other elements either as additional components of macromolecules (for example, phosphorus, sulfur, and selenium), as cofactors required for the structural integrity (such as zinc) or enzymatic activity (such as copper and iron) of proteins, or as second messengers in cellular signal transduction (such as calcium). Because of the many important roles these elements play in cellular biochemistry, efficient mechanisms are required to obtain these nutrients from the environment, utilize or store them within intracellular organelles, and regulate their intracellular abundance to prevent overaccumulation and resultant toxicity. Identifying the molecular components of these mechanisms is a critical step toward a complete understanding of the nutritional aspects and toxicity of these elements. In addition, such information will be important as we attempt to genetically engineer plants and other organisms that are capable of removing toxic elements from the environment to remediate polluted sites (bioremediation).

The yeast *Saccharomyces cerevisiae *has been a useful model organism for the study of many different fundamental cellular processes, including the uptake, metabolism, and homeostatic control of mineral nutrients and trace elements. The usefulness of yeast for genome-wide studies of nutrient homeostasis has markedly increased with the recent completion of the *Saccharomyces *Genome Deletion Project [1]. This effort resulted in a collection of mutant strains disrupted in most of the approximately 6,000 genes in the yeast genome. This strain collection provides a unique resource for the analysis of gene function in a model eukaryotic cell.

Many studies of yeast have focused on the molecular mechanisms relevant to the utilization of nutrients [2-5]. The great majority of these studies have focused on the metabolism of specific nutrients without considering the effects of these systems on other elements. Thus, despite our growing understanding of the mechanisms controlling specific nutrients, the individual genes and gene networks that influence the acquisition and utilization of multiple elements remain largely unknown. To address this question, we have combined the genomic technologies provided by the *Saccharomyces *Genome Deletion collection with spectroscopic methods for the simultaneous analysis of multiple mineral nutrients accumulated by cells. The method used here, inductively coupled plasma-atomic emission spectroscopy (ICP-AES), can detect a broad range of elements simultaneously in a single assay [6]. The high sensitivity and dynamic range of this technology allows for the accurate quantitative measurement of element levels in small sample volumes.

Using ICP-AES, we have defined the elemental profile of wild-type yeast cells grown under standardized laboratory conditions. We refer to this profile as the yeast 'ionome', which expands on the previous concept of the 'metallome' to include several nonmetals [7-9]. The levels of 13 elements were assayed: calcium, cobalt, copper, iron, magnesium, manganese, nickel, phosphorus, potassium, selenium, sodium, sulfur, and zinc. We then determined the ionome profiles for a collection of over 4,000 different yeast mutants. The results of this study provide insights into the cellular systems controlling the homeostasis of multiple nutrients and provide new data for the functional characterization of as yet unstudied yeast genes.

## Results and discussion

### Characterizing the ionome of wild-type yeast cells

In this study, we used ICP-AES to simultaneously determine the levels of 13 different elements accumulated in yeast cells. Rich yeast extract-peptone-dextrose (YPD) medium was supplemented with several elements (calcium, cobalt, copper, manganese, nickel, selenium, zinc) to levels sufficient to facilitate their detection in cell extracts by ICP-AES (see Materials and methods). Boron and molybdenum were also added to the medium but these elements did not accumulate to sufficient levels to allow their detection by our methods. Furthermore, while neither nickel nor selenium is known to be required for yeast cell growth, many organisms use these elements for a variety of roles. Therefore, they were included in this analysis in the hope of better understanding the factors affecting their accumulation. In no case did the supplemented concentration of these elements exceed 10% of the minimal growth inhibitory concentration determined for this wild-type strain of yeast (data not shown).

**Figure 1:**
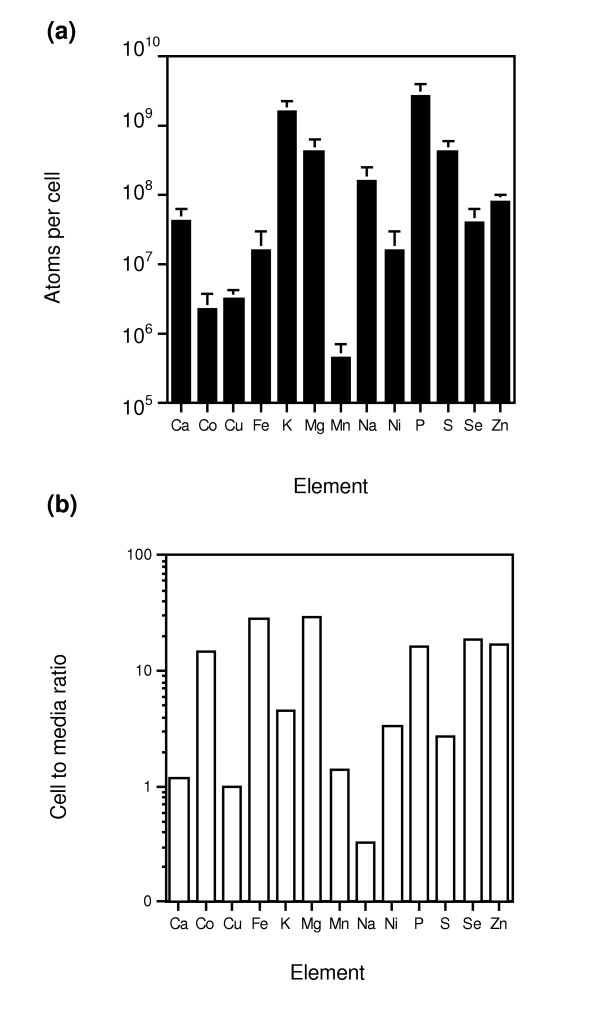
Characterization of the wild-type yeast ionome. **(a) **Wild-type BY4743 cells were grown in rich yeast extract-peptone-dextrose (YPD) + mineral supplements to post-diauxic-shift phase, harvested, digested with HNO_3_, and then analyzed for the levels of the indicated elements. Mean values are shown and the error bars indicate 1 standard deviation (*n *= 40). **(b) **The element content of the supplemented growth medium was also assayed (*n *= 6). The ratio of cell concentration, calculated from the data in panel (a) and assuming homogeneous distribution in the cell, to medium concentration is plotted.

Cells were grown to the post-diauxic-shift phase before harvesting. The cells were then collected by filtration and thoroughly washed to remove extracellular elements, and the organic material was then digested by overnight incubation in concentrated nitric acid before ICP-AES analysis. The 13-element ionome profile determined for wild-type cells is shown in Figure 1a. The minerals detected in our analysis accumulated to levels spanning almost four orders of magnitude, demonstrating the broad range over which these elements are found in living cells. Those elements that accumulated to the lowest levels were the trace elements manganese, cobalt, and copper (0.5 to 3 × 10^6 ^atoms per cell). Those accumulating to the highest levels were the macronutrients potassium and phosphorus (1.6 to 2.8 × 10^9 ^atoms per cell). The level of accumulation for many of these elements was very different from that observed previously for *Escherichia coli *grown in rich LB (Luria-Bertani) medium [7]. When converted to molar concentrations to adjust for the differences between bacterial and yeast cell volume and assuming homogeneous intracellular distributions, the accumulated levels of copper, potassium, magnesium, and manganese were similar to the levels in *E. coli*, whereas others, such as calcium, iron, and zinc, accumulated in yeast to 10-fold higher levels. Some of these differences may reflect the ability of eukaryotic cells to accumulate high levels of these elements within intracellular organelles that are not present in prokaryotes. Previous studies have indicated that yeast cells store many mineral nutrients within intracellular organelles [10-13].

We also compared the levels of these elements within cells with the corresponding levels in the growth medium (Figure 1b). Elements such as calcium, copper, and manganese accumulated to similar molar concentrations relative to the medium used for this study. As expected, sodium was largely excluded, with cells showing only 30% of media levels. In contrast, cobalt, iron, potassium, magnesium, phosphorus, sulfur, selenium, and zinc accumulated in cells to 3 to 30 times the level in the external environment, an observation consistent with the ability of cells to concentrate these elements intracellularly.

### Analysis of yeast deletion mutants for effects on the ionome profile

To identify yeast genes critical to the homeostatic control of these elements, we determined the ionome profile of mutants generated by the *Saccharomyces *Genome Deletion Project. Approximately 25% of the total number of yeast genes (approximately 6,000) are either essential for viability under our growth conditions or had not yet been generated by the deletion project at the inception of this project. Therefore, we did not assay these strains. As a result, we analyzed a total of 4,385 different yeast mutants for their effects on the yeast ionome. To facilitate a genome-wide analysis, all of these strains were subjected to a high-throughput 'first-pass' ionome profile determination in which cells from a single culture of each mutant strain were assayed (see Additional data file 1 for a complete list of all strains tested). Of those 4,385 yeast mutants, 773 (18%) were identified as showing a twofold or greater difference for at least one element relative to triplicate wild-type controls prepared alongside each set of mutant samples. These 773 strains were then subjected to a 'second-pass' analysis of three independent cultures for each strain. A total of 233 strains were then identified that showed differences exceeding 3 standard deviations from the wild-type mean for at least one element in their respective profiles. These 233 strains were then analyzed in a 'third-pass' analysis of six independent cultures for each. Through this process, a total of 212 strains were identified as having mutations that cause reproducible effects on the yeast ionome, judged here as mean values increasing or decreasing by more than 2.5 standard deviations of the wild-type mean. The high ratio of strains showing reproducible effects in the second- and third-pass experiments (212/233 or 91%) indicates that few false positives are likely to be present in the final list of mutants identified as having ionome changes. Including cultures of wild-type cells assayed as controls, our results are based on the ICP-AES analysis of over 10,000 independent cultures.

**Figure 2:**
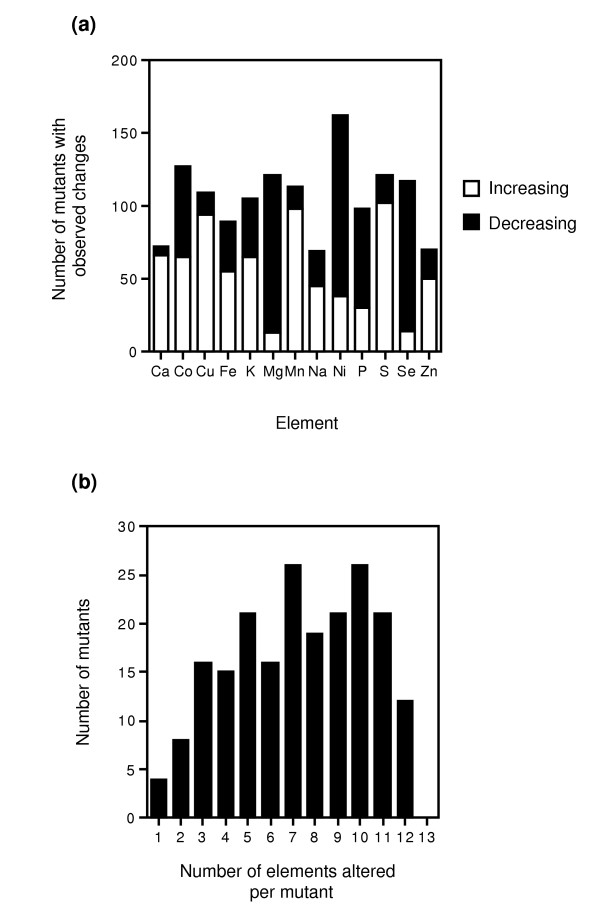
Overview of the effects of mutations on element content. **(a) **Number of mutants showing increases (open bars) and decreases (filled bars) for each element. **(b) **Number of mutants showing one or more changes in their ionome profiles.

The specific mutations leading to alterations in the level of one or more element are listed in Additional data file 2. An analysis of the effects of these mutations on the accumulation of specific elements revealed remarkable differences among them (Figure 2a). First, sodium and zinc showed the fewest number of mutants with alterations in their levels (69 and 70 of 212 total mutants, respectively). In contrast, nickel levels were altered in the most strains (162 of 212). When these effects were examined in more detail, the elements could be divided into three distinct groups. First, for elements such as cobalt, iron, and potassium, approximately equal numbers of mutants showed increases and decreases in element accumulation. In marked contrast, the results for calcium, copper, manganese, sulfur, and zinc were dominated by mutants showing increased mineral levels, whereas decreased levels were most frequently observed for magnesium, nickel, and selenium.

The numbers of mutants affected for each element represented in Figure 2a add up to considerably more than the 212 total strains identified in the analysis. This observation demonstrates an additional important point arising from these data. Most of these mutations are very pleiotropic in their effects on the ionome profiles; that is, more than one element was frequently altered for a given mutant. This pleiotropy is also clear when the number of elements affected per mutant is plotted versus the number of mutants (Figure 2b). The number of elements altered per strain ranged from as few as 1 element (4 mutants) to as many as 12 of the 13 elements we measured (12 mutants). A peak distribution observed in our experiments was around 7 to 10 elements affected per mutant.

### Functional classification of mutations that alter the yeast ionome

**Figure 3:**
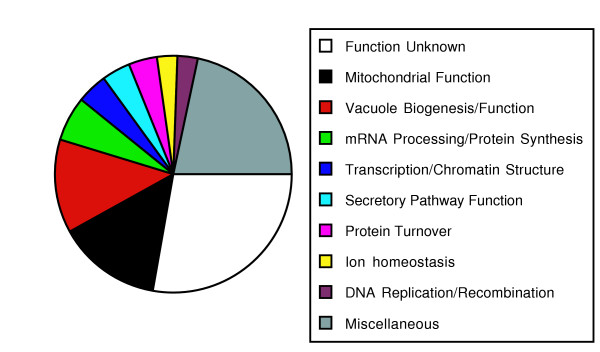
Functional classes of genes identified by ionome profiling of their corresponding mutants. The number of genes identified in each functional class is represented. See Additional data file 3 for a complete list of the specific genes in each functional category.

The genes altered in the 212 mutant strains were grouped into 25 broad functional classes. An analysis of the distribution of the mutant strains among these functional classes is shown in Figure 3 and the specific genes in all groups are listed in Additional data file 3. The largest class had mutations in genes encoding proteins of unknown function, representing approximately 25% (59) of the mutants identified. This percentage reflects the relative frequency of genes in the entire yeast genome that remain uncharacterized. The two largest classes of mutations affecting proteins of known function were those with effects on vacuole biogenesis and function (27 mutants) (Table 1) and those involved in mitochondrial function (30 mutants) (Table 2). Classes containing fewer mutants included those affecting proteins involved in secretory pathway function (8). Thus, the largest percentage of genes identified (65 of 212 genes or 31%) are involved in the biogenesis or function of intracellular organelles. This result emphasizes the importance of these compartments in ion homeostasis. Other functional classes include genes involved in mRNA processing and protein synthesis (13) and transcription/chromatin structure (9). These classes of mutants are likely to cause changes in mineral content through indirect effects on gene expression and/or protein abundance. Surprisingly, genes known to be specifically involved in ion homeostasis accounted for only 3% (6/212) of the genes identified.

### Effects of mutations disrupting organellar function on the ionome profile

**Figure 4:**
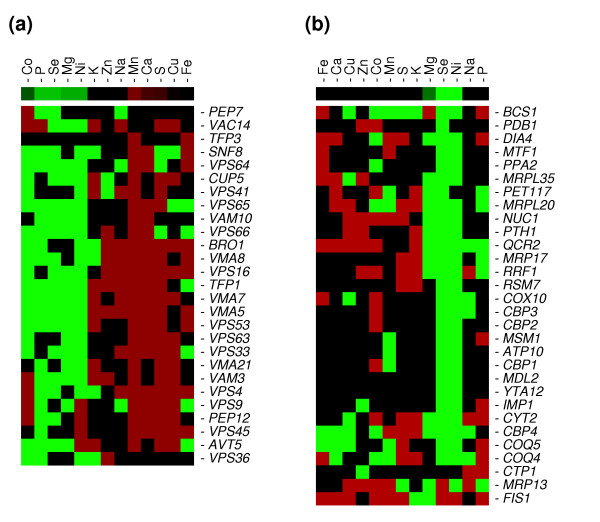
Mutants within functional categories show similar ionome phenotypes. The effects of mutations altering **(a) **vacuolar or **(b) **mitochondrial function on the ionome profile are shown. Elements are listed along the horizontal axis and the genes affected are listed along the vertical axis. Increases greater than 2.5 standard deviations of the wild-type means are shown in red and decreases greater than 2.5 standard deviations are shown in green. The bars at the top represent the consensus for each group of genes. This figure was generated using TreeView software.

The major role of organelles in controlling the ionome profiles warranted closer examination. As shown in Figure 4a, many mutants defective for vacuolar biogenesis and/or function caused increased accumulation of manganese, calcium, sulfur, and copper as well as decreases in cobalt, phosphorus, selenium, magnesium, and nickel. These were among the most pleiotropic mutations identified. The 27 vacuole-related mutants identified affected genes involved in many aspects of vacuolar function. First, six of these mutants were altered in genes encoding subunits of the vacuolar H^+^-ATPase (for example, *CUP5*, *TFP1*) (Table 1). These mutants have normal vacuole morphologies but lack the ability to acidify the organelle [14]. It was initially surprising that only a subset of V-ATPase mutants were identified in our screen, given that mutations in these genes are very likely to cause the same phenotypes. An examination of the ionomics dataset indicated that about half of the V-ATPase subunit mutants failed to meet the twofold cutoff criterion used in our first-pass analysis to identify strains for reanalysis. This observation suggested that the high stringency of this cutoff value was the main reason these genes were not included in our final list of mutants. Confirming this hypothesis, we reassayed eleven V-ATPase subunit mutants (*n *= 6) and found good accord among them. For example, 9 of the 11 mutants showed increased manganese and 8 of the 11 strains had significantly increased copper and decreased selenium (Additional data file 4).

Several mutants affecting vacuolar biogenesis were also identified. Previous studies of vacuolar protein sorting in yeast resulted in the identification of six classes, designated A through F, of mutants affecting this process [15,16]. These mutant classes exhibit a number of different vacuolar morphologies. For example, mutants of class A have normal-appearing vacuoles but show defects in protein sorting. Class B mutants have fragmented vacuolar morphologies, while class C mutants lack any recognizable vacuolar structure. Class D mutants have defects in vacuolar inheritance, resulting in daughter cells with a class C appearance, while class E mutants accumulate vacuolar proteins in the prevacuolar compartment because of defects in membrane trafficking from this compartment to the vacuole or the Golgi apparatus. Mutants of the final group, class F, have both normal-appearing vacuoles and fragmented vacuoles similar to those of class B mutants. Vacuolar mutants of four of these six classes were found to affect the ionome (Table 1). No mutants of either class A or F were identified, suggesting that the normal-appearing vacuoles in mutants of these classes are capable of maintaining the wild-type ionome profile. In addition to the 27 vacuolar mutants, several of the mutants with altered secretory pathway function (for example, *RIC1*, *YPT6*, *COG7*, *COG8*) showed similar profiles to the vacuolar mutants, suggesting that the effects of these mutations are due to indirect disruption of vacuolar function.

Thirty genes required for mitochondrial function were also identified (Table 2). These include genes required for mitochondrial transcription and protein synthesis (for example, *MTF1*, *MRPL20*, *MRPL35*), mitochondrial mRNA processing (for example, *CBP1*, *CBP2*), electron transport chain function (for example, *COX10*, *CYT2*, *COQ4*, *COQ5*), and oxidative phosphorylation (for example, *ATP10*). These mutants share common disruption of selenium and nickel accumulation, with the levels of both decreasing (Figure 4b). These effects were clearly distinguishable from the effects of vacuolar mutants that showed changes in other minerals in addition to nickel and selenium.

Finally, mutants disrupted for five genes involved in endocytosis (*CLC1*, *SAC6*, *RVS161*, *RVS167*, and *YPK1*) were also isolated. All five mutants showed increases in both calcium and copper accumulation. This result is consistent with the likely contribution of endocytosis to downregulating yeast copper uptake transporters [17] and suggests that calcium accumulation may be regulated in a similar fashion. To our knowledge, this potential mechanism of calcium homeostasis has not been tested experimentally.

### The interrelationships between different elements in the yeast ionome

**Figure 5:**
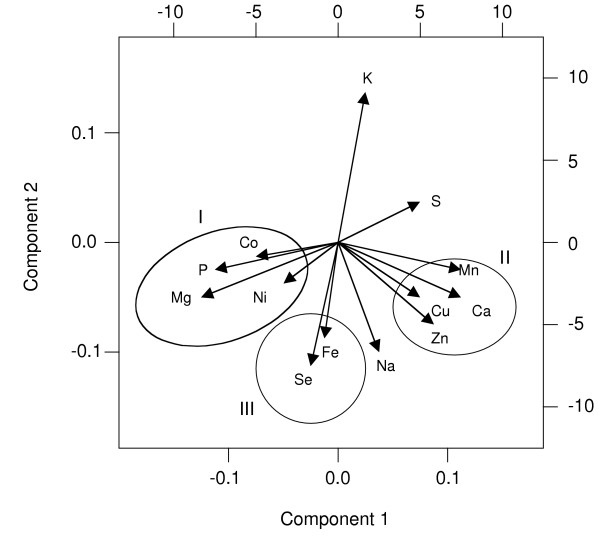
Biplot representation of the ionome results. The length of each eigenvector is proportional to the variance in the data for that element. The angle between eigenvectors represents correlations among different elements. Three groups of elements (circled, and denoted I, II, and III) show strong positive correlations.

The similar effects of mutations in particular functional categories suggests that the homeostatic mechanisms that control the levels of different elements are interconnected. For example, mutants defective for vacuolar function show similar effects on several elements. This point was further emphasized when the entire third-pass ionome dataset was analyzed by principal-component analysis [18]. Both positive and negative correlations among elements are readily detected by this analysis and the results are presented as a biplot graph in Figure 5. The length of each eigenvector arrow reflects the variance in the data for each element. Thus, considering the configuration of the 13 elements depicted in Figure 5, it is evident that the largest variance is seen for potassium, while cobalt and nickel show the smallest variances. The biplot representation also displays the relationships among elements. The angles between positively correlated eigenvectors approach 0° while those between negative correlations approach 180° on the biplot representation. Elements showing no correlation have 90° eigenvector angles. Significantly, several of the elements cluster into one of three positively correlated groupings. In group I, magnesium, phosphorus, cobalt, and nickel are found to correlate in a large number of mutant strains. In addition, the elements in group I show a strong negative correlation with the effects of these mutations on sulfur levels. In group II, calcium, manganese, copper, and zinc show a strong correlation with each other, while group III includes iron and selenium. Group III elements also show a strong negative correlation with potassium. Some of the possible molecular explanations underlying these relationships will be considered below.

To our knowledge, this genomic analysis of ionome profiles is only the second of its kind, the first being the analysis of random mutants in *Arabidopsis *[8]. This yeast study has the added benefit of using a collection of already defined mutant strains. The results from yeast differ from the plant study in two significant ways. First, a greater degree of pleiotropy was observed among the yeast mutants than in plants. As shown in Figure 2b, the number of elements affected per strain peaked at around 7 to 10. In contrast, the peak among the plant mutants was at three elements altered per plant line. The second major difference is in the effects of mutations on particular elements. As shown in Figure 2a, the results for some elements are dominated by mutants showing either increases or decreases in their accumulation. While similar trends were observed among the plant results for some elements (such as copper), others differed markedly. For example, while most mutants in yeast affecting calcium caused increased accumulation, the majority of plant mutants had the opposite effect. Magnesium, phosphorus, nickel, and selenium show similarly divergent results. The dissimilar results obtained with yeast and plants may reflect fundamental differences in the cellular metabolism of these elements or, more likely, differences in element homeostatic mechanisms at work in single-celled versus multicellular organisms.

We found that mutations in 3% to 4% of the total genes in the yeast genome caused reproducible effects on the ionome under the growth conditions we used in this study. A similar recovery rate was obtained in the *Arabidopsis *study [8]. It was initially surprising that only 6 of the 212 yeast genes identified were previously determined to play specific roles in mineral homeostasis either as transporters or as transcription factors controlling expression of transporters and other genes. These genes are *SMF3*, *CCC1*, *GEF1*, *SPF1*, *RCS1 *(*AFT1*), and *ROX1*. *SMF3 *and *CCC1 *encode metal ion transporters in the vacuolar membrane [11,19]. *GEF1 *encodes a chloride channel in the Golgi apparatus that is involved in assembly of a functional iron uptake system [20], and *SPF1 *encodes a P-type ATPase in the secretory pathway whose substrate is unknown but likely to be an inorganic ion, perhaps Ca^2+ ^[21]. Aft1 controls genes involved in iron uptake and metabolism, while Rox1 represses genes under aerobic conditions. At least one Rox1 target gene, *FET4*, is involved in metal ion uptake [22,23]. Mutants affecting many genes known to play roles in the homeostasis of these elements under certain conditions were included in our analysis. These included transporters involved in calcium (Cch1, Pmr1, Vcx1, Pmc1), cobalt (Cot1), copper (Ctr1, Ccc2), iron (Fet3, Ftr1, Smf3), magnesium (Alr1, Mrs2, Lpe10), manganese (Smf1, Pmr1, Atx2), phosphorus (Pho87, Pho88, Pho89), potassium (Trk1, Trk2, Tok1), sodium (Nhx1, Nha1), sulfur (Sul1), and zinc (Zrt1, Zrt3, Zrc1). The remarkably small number of such genes in our final list of mutants probably represents the redundancy of systems involved in the uptake and intracellular distribution of minerals. The cells in these cultures were grown under nutrient-rich conditions where multiple systems are likely to mediate these processes. For example, at least four different zinc uptake systems (Zrt1, Zrt2, Fet4, and one unknown system) are present in yeast [23-25] and loss of any one system fails to exert a major effect on the overall zinc accumulation under these conditions because of the compensatory control of the other pathways. As a further example, vacuolar storage of zinc requires the Zrc1 and Cot1 transporters, but mutation of either single gene has very little effect on zinc accumulation in the vacuole [12].

By far the largest groups of previously characterized genes that we identified were those involved in the function of the vacuole or the mitochondria. This observation highlights the importance of these compartments in maintaining mineral homeostasis. Vacuolar mutants were found to frequently show increases in manganese, calcium, sulfur, and copper as well as decreases in cobalt, phosphorus, selenium, magnesium, and nickel accumulation. The effects of these mutants on nickel and selenium accumulation may be explained by the current hypotheses that both of these elements are detoxified in the vacuole [26,27]. Failure to accumulate nickel and selenium in the vacuole may increase their cytosolic concentrations and thereby inhibit further uptake. The vacuole has also been previously implicated in the intracellular storage of phosphorus and magnesium, and our results support those of previous studies. Phosphorus is stored in great abundance in the vacuole as polyphosphate: long chains of phosphate groups linked by phosphoanhydride bonds. This material, which can accumulate to ≥10% of the dry weight of a yeast cell, has been proposed to bind Mg^2+ ^to facilitate its storage in the vacuole [28]. This scenario provides a plausible explanation for the effects of vacuolar mutants on both phosphorus and magnesium accumulation; that is, the decrease in polyphosphate accumulation decreases the binding capacity for Mg^2+ ^in the vacuole lumen. Consistent with this role, we found here that mutations that disrupt polyphosphate accumulation [29], namely *vtc1/phm4 *and *vtc4/phm3*, also reduce magnesium accumulation.

Given the ability of vacuolar polyphosphate to bind other metal ions such as Zn^2+^, it was predicted that mutations in vacuolar function would also disrupt accumulation of other metals [30]. The vacuole has been implicated as a major storage site for excess intracellular zinc [12,31]. However, no strong correlation was observed between mutants affecting vacuolar biogenesis and/or function and zinc levels in this study, indicating that polyphosphate may not be required for zinc storage. Alternatively, while disruption of the vacuole may indeed reduce vacuolar zinc storage, other compartments (for example, mitochondria) may then accumulate the excess zinc and maintain a consistent total cellular content [32]. It is also intriguing to note that disruption of the vacuole does not consistently alter the accumulation of iron, which has recently been proposed to be stored there [11]. Accumulation in other sites as proposed above for zinc may be involved here as well. Mitochondria have been found to be a site for iron accumulation under certain conditions [33,34]. Thus, impairment of vacuolar iron storage may lead to increases in mitochondrial iron under our culture conditions.

In addition to these other elements, calcium and manganese are also thought to accumulate in the yeast vacuole and are probably bound by polyphosphate [13,35,36]. However, mutants with altered vacuolar function showed consistent increases in the accumulation of these metals. While surprising, this observation is not without precedent. Miseta *et al*. noted previously that mutations in *VPS33*, a class C vacuolar protein-sorting gene, caused elevated cellular calcium accumulation [36]. These authors attributed this increase to an activation of the Pmr1 Ca^2+^/Mn^2+^-transporting ATPase located in the Golgi apparatus and subsequent calcium hyperaccumulation in that compartment. Given the ability of Pmr1 to transport both Ca^2+ ^and Mn^2+^, this scenario may also explain the effects of vacuole disruption on total manganese accumulation observed here. Our results extend those previous observations by demonstrating that mutations that disrupt vacuolar acidification without disrupting vacuolar morphology also have this effect. Therefore, it is likely that hyperaccumulation of calcium and manganese in these mutants arises from the downstream effects of failing to store these ions in the vacuole. In a previous study, Ramsay and Gadd observed that mutants disrupted for vacuolar acidification had reduced manganese accumulation, whereas the levels of manganese increased in our experiments [13]. The treatment conditions were very different in these two studies. The previous study used 1 mM manganese while our medium contained only 11 μM manganese, and this difference may explain the opposite results. Thus, the vacuole may play a greater role in manganese storage under extremely high manganese conditions.

Another intriguing observation of this study is the strong negative correlation between the elements in group I (magnesium, phosphorus, nickel, cobalt) and sulfur (Fig. 5). One major driving force for this inverse correlation may be the role of the vacuole in sulfur homeostasis as well as for magnesium and phosphorus, for example, as noted above. Strains carrying mutations in 21 of the 27 genes identified in our study that are involved in vacuolar biogenesis and function showed marked increases in sulfur levels. The underlying molecular mechanism for this increase is currently unclear. One possible explanation is the potential role of the vacuole in sulfur storage. *S*-adenosylmethionine (AdoMet) is one of the major organic sulfur compounds in cells. Intracellular levels of AdoMet are approximately 1 mM [37], with about 70% of the total accumulating in the vacuole [38]. Given the effects of vacuolar mutations on other elements such as magnesium and phosphorus, we would have predicted *a priori* that the total levels of sulfur would decrease in these vacuolar mutant cells. Surprisingly, the effects are just the opposite: vacuolar mutants accumulate more sulfur than do wild-type cells. This effect was observed in a previous report where *vps33 *mutants were isolated because they hyperaccumulated AdoMet [39]. Because the transcriptional control of methionine biosynthetic genes are responsive to intracellular AdoMet levels [40], this hyperaccumulation led to the inappropriate repression of methionine biosynthesis and, therefore, methionine auxotrophy. Based on the analysis of the methionine auxotrophy phenotype, it was concluded that the disruption in sulfur homeostasis was limited to vacuolar mutants that eliminated the vacuolar structure (that is, class C mutants) and did not occur in mutants with lesser defects in vacuolar function [39]. In contrast, our results indicate that sulfur homeostasis is disrupted even in mutants that retain vacuolar structure but are simply unable to acidify the organelle (*vma5*, *vma7*, *tfp1*). The question still remains how disruption of vacuolar function leads to increased sulfur accumulation. It is conceivable that sulfur homeostasis is mediated in part by a signal of AdoMet storage in the vacuole. Loss of that signal due to vacuolar disruption might then lead to increased sulfur accumulation elsewhere in the cell.

Several other novel relationships between elements were also observed in this study. For example, iron and selenium show a strong positive correlation with each other and also a strong negative correlation with potassium accumulation. Genes showing this profile include those functioning in vacuolar function (*TFP1*, *AVT5*), secretary pathway function (*COG7*, *COG8*, *RIC1*), protein synthesis (*RPL22A*, *RPL23A*, *RPL27A*), and ion homeostasis (*SPF1*, *ROX1*). Given the diverse processes represented in this group of genes, future studies will be required to discover the mechanism(s) underlying this correlation.

The data obtained in this study are likely to be useful in assigning function to genes that have not yet been characterized. Among the 212 genes identified are 59 of unknown function. Many of these mutants show ionome profile patterns consistent with other profiles observed in the dataset. For example, mutants disrupted for 11 genes (*YGL260W*, *YGR122W*, *YGR206W*, *YHL005C*, *YHL029C*, *YHR033W*, *YIR024C*, *YKL075C*, *YKR035C*, *YMR066W*, and *YMR098C*) showed increased accumulation of nickel and selenium without the broader effects observed in vacuolar mutants. This profile is similar to that observed among mutants with disrupted mitochondrial function. Therefore, these genes may perform some role in mitochondria. Consistent with this prediction, three of their protein products have been tentatively localized to mitochondria by a genome-wide protein localization project (*YIR024C*, *YMR066W*, *YMR098C*) [41]. In addition, mutants disrupted for these three and a fourth gene (*YHL005C*) in this group not yet localized grow poorly on carbon sources requiring respiration [42].

**Figure 6:**
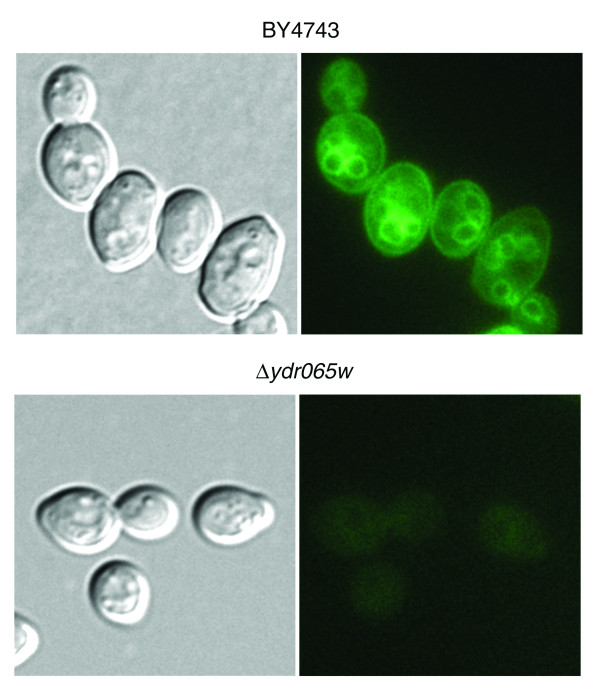
Δ*ydr065w *mutants are defective for vacuolar acidification. Wild-type (BY4743) and BY4743 Δ*ydr065w *cells were harvested in exponential phase, incubated with LysoSensor Green DND-189, and then examined by differential interference contrast (DIC) (left panel) and fluorescence (right panel) microscopy. Failure to accumulate the fluorophore indicates defective vacuolar acidification. Intact vacuoles in the mutant cells are apparent in the DIC image.

In addition, ionome profiles similar to vacuole-defective mutants are also displayed by mutants disrupted in six uncharacterized genes (*YDR065W*, *YDR220C*, *YGL220W*, *YGL226W*, *YKL171W*, *YOR331C*). Thus, the encoded proteins are likely to be involved in vacuolar biogenesis or function. To test this hypothesis, the ability of the Δ*ydr065w *mutant to acidify its vacuole was assayed using LysoSensor Green DND-189 (Molecular Probes, Eugene, OR, USA). Accumulation of this fluorophore in the vacuolar membrane is dependent on the lumenal acidity of the compartment. As shown in Figure 6, the Δ*ydr065w *mutant failed to accumulate LysoSensor Green DND-189, indicating a severe disruption of vacuolar acidification. Similar results were also obtained with quinacrine (data not shown), another marker of vacuolar acidification. These results clearly demonstrate that the ionomics data provide important clues about the function of uncharacterized genes.

## Conclusion

In this study, we used a genome-wide approach to identify genes that control the yeast ionome. With the application of ICP-AES, we determined the elemental profile of mutants defective in over 4,000 different yeast genes. Of these, 212 mutant strains were identified that showed reproducible changes in their ionome profiles. The majority of these mutants had pleiotropic effects with changes in the levels of multiple elements. Both positive and negative correlations were observed among groups of elements, thereby highlighting previously unsuspected relationships between elements. Mutants in certain functional categories, such as those with disrupted vacuolar or mitochondrial function, showed related ionome profile changes. We show that these results can then be used to develop hypotheses regarding the functions of previously uncharacterized genes. It is noteworthy that our ionomics analysis used post-diauxic-shift cells grown in a rich medium. Different results would most likely be obtained using exponential-phase cells and/or cells grown in minimal media or with other carbon sources. This ionomics approach provided new information about the mechanisms controlling mineral accumulation in yeast. Given that *S. cerevisiae *has served as such a useful model for the study of many different processes, including mineral homeostasis, we predict that insights ultimately gained from this type of analysis will also aid in our understanding of how plant and animal cells control these processes at the cellular and perhaps even organismal levels.

## Materials and methods

### Yeast strains analyzed

The mutants analyzed were prepared by the *Saccharomyces *Genome Deletion Project [1] and were purchased from Open Biosystems (Huntsville, AL, USA). The method used to generate this collection was a polymerase chain reaction based deletion strategy to generate a complete deletion of each of the open reading frames in the yeast genome. As part of the deletion process, each gene was replaced with the KanMX module, which confers resistance to G418. We analyzed the homozygous diploid collection generated in strain BY4743, whose full genotype is *MATa/MAT*α *his3/his3 leu2/leu2 ura3/ura3 lys2/+ met15/+*. Nearly all open reading frames larger than 100 codons were disrupted in this collection. Many yeast genes are essential for growth in rich medium, so the corresponding strains were not included in our analysis. In addition, closely related genes (for example, *ENA1-5*) with ≥97% similarity were also not tested, because mutants in these genes were not generated by the deletion project consortium.

### Culture conditions

Cells were recovered from frozen stocks and streaked for colonies on agar plates containing YPD (1% yeast extract, 2% peptone, 2% glucose) + 200 μg/ml G418 (Sigma, St Louis, MO, USA). A single colony from each plate was then inoculated into 5 ml of YPD + 1/100 volume of a 100 × mineral supplement stock (Table 3). The effects of metal supplementation on accumulation by wild-type cells is presented in Additional data file 5. In later experiments with multiple replicates, either three or six separate colonies from each strain were used for inoculations. The cells were grown with aeration at 30°C to post-diauxic-shift phase (≥7.5 × 10^7 ^cells/ml). For most strains, this phase was reached after 2 days of culturing. Slower-growing strains were harvested at similar cell densities after longer periods of incubation. In preliminary studies, we found that exponential-phase cells and post-diauxic-shift cells accumulate different levels of some minerals. For example, accumulation of iron, manganese, and zinc doubles in post-diauxic shift phase cells, while copper, nickel, and selenium levels increase more than 10-fold (data not shown). Post-diauxic-shift cells were used for this analysis because large numbers of cells could be obtained in smaller and more manageable culture volumes. Therefore, when considering the effects of mutations on the yeast ionome, it should be noted that different results may be obtained with cells harvested in exponential phase. No mutants assayed showed ionome profiles similar to exponential-phase cells. Three wild-type control cultures were included in each first-pass (*n *= 1) and second-pass (*n *= 3) experiment for use as references. Six wild-type cultures were included in each third-pass (*n *= 6) experiment.

### Sample processing and ICP-AES analysis

The same lots of all medium components (such as yeast extract, peptone) were used throughout this study, to maintain consistent growth conditions. Culture volumes of 2.5 ml were collected by vacuum filtration using Isopore membrane filters (1.2 μm pore size) (Fisher Scientific, Pittsburgh, PA, USA). Cells were then washed three times with 5 ml of 1 μM ethylenediaminetetraacetic acid disodium salt solution, pH 8.0, by vacuum filtration followed by three washes with 5 ml each of distilled, deionized H_2_O. Pilot studies indicated that these conditions efficiently remove unbound elements (data not shown). The filters were then placed in screw-top microcentrifuge tubes, and 500 μl 30% HNO_3 _was added. The samples were digested overnight in a 65°C water bath. Afterward, 500 μl of distilled, deionized H_2_O was added, the samples were vortexed briefly, and the filters were removed. The cell digests were then centrifuged for 10 min at 12,000 × *g *and the supernatants were transferred to new tubes. ICP-AES analysis was performed with a Varian Vista ICP-AES (Varian Inc, Palo Alto, CA, USA) with a three-channel peristaltic pump.

The genes altered in the 212 mutant strains were grouped into 25 broad functional classes based on information available in the literature, the *Saccharomyces *Genome Database [43] and the Comprehensive Yeast Genome Database [44].

### Data scaling and normalization

The scaling and normalization process was based on the concept that the wild-type samples differ only in total cell mass. They can therefore be brought to a common scale using a scale factor equal to the median value of the ratio of each element to a common standard. This scale factor can then be used to normalize the mutant data in the same experiment. The specific subset of elements used for scaling is given by *E *= {*K*, *Ca*, *Mg*, *Mn*, *P*, *S*, *Zn*}. The common standard used for normalization is the average concentration of each element over the set of wild-type samples within the experiment (*n *= *6*). These standard values *a*, for each element *j *are given by:



where *x*_*ij *_is the concentration of metal *j *in replicate *i*. The scale factor, *w*_*i*_, for a particular replicate sample *i *is then given by:



The scaled average concentrations, , of each element *j *in the wild-type samples are given by:

 with standard deviation .

The scaled concentration of the elements in the mutant samples, , relative to wild type, is then calculated in a similar way, with scale factors for each replicate, , calculated with respect to the average values of the wild-type samples in the same experimental set, and *z *scores (number of standard deviations from the wild-type means) were calculated relative to these same wild-type samples.



 with standard deviation 



The *z *scores so obtained were then transformed to (1, 0, -1) depending on whether the values were greater than 2.5, from 2.5 to -2.5, or less than -2.5. This was then used for higher level analyses.

### Principal-component analysis and biplot PCA

Principal-component analysis (PCA) and biplot PCA were used to characterize the structure of correlations within the ionome data and biplot [18] to present the results of the PCA. Briefly, a biplot is a graphical display of a matrix *M *= (*m*_*ij*_) of *n *rows and *m *columns, using markers *r*_1_, *r*_2_,..., *r*_*n *_for its rows and markers *c*_1_, *c*_2_,..., *c*_*m *_for its columns. These markers are chosen in such a way that the inner product *r*_*i*_^T ^*c*_*i *_represents *m*_*ij*_, the *i*, *jth *element of *M*. 'bi' in the biplot indicates a joint display of row and columns of the matrix *M*. The rows for the ionome matrix correspond to yeast knockouts and the columns are the ions. The dimensionality of the matrix for the ionome is 212 × 13. The row markers correspond to genes knocked out (not shown in the figure) and the arrows or column markers represent ions. The length of the arrow represents the variances of the different ions and the angle represents their correlation.

### LysoSensor Green DND-189 labeling

Assessment of vacuolar acidification was performed with LysoSensor Green DND-189 as previously described [45].

## Additional data files

The following additional data are available with the online version of this paper. Additional data file 1 is an Excel file summarizing the mutants analyzed. Additional data file 2 is an Excel file showing the effects of mutations in yeast genes on their respective ionome profiles. Additional data file 3 is an Excel table showing the functional classifications of the 212 strains showing reproducible effects. Additional data file 4 is an Excel file showing ionome analysis of mutants disrupted for V-ATPase subunit genes. Additional data file 5 is an Excel file showing the effects of metal supplementation on accumulation by wild-type cells.

## Supplementary Material

Additional data file 1 The mutant strains assayed in the first-pass (*n *= 1), second-pass (*n *= 3), and third-pass (*n *= 6) inductively coupled plasma-atomic emission spectroscopy (ICP-AES) analyses are listed. Genes identified by genome sequencing or other studies that were not analyzed are also listed. Click here for file

Additional data file 2 Shown are the results of the third-pass (*n *= 6) inductively coupled plasma-atomic emission spectroscopy (ICP-AES) analysis. Sheet 1: Normalized concentrations in parts per million (ppm) for each of the 13 elements assayed. Sheet 2: *z *scores are reported for each mutant and each element. These values are the number of standard deviations that the mutant value differed from the mean of the wild-type samples included in that experiment. Sheet 3: The *z *score data from Sheet 2 were converted into trinary data. A value of 1 was assigned if the *z *score was ≥2.5 and -1 if the *z *score was ≤-2.5. Values of 0 were assigned for values that fell between those boundaries. Click here for file

Additional data file 3 The descriptions of gene function were obtained from the *Saccharomyces *Genome Database [43]. Click here for file

Additional data file 4 Shown are the results of an inductively coupled plasma-atomic emission spectroscopy (ICP-AES) analysis (*n *= 6) of V-ATPase subunit mutants grown in yeast extract-peptone-dextrose (YPD) medium with metal supplements. Normalized accumulation is reported in parts per million (ppm) and *z *scores are reported for each element. Calcium values from this experiment are not reported due to a high background of this element in these particular samples. Click here for file

Additional data file 5 Shown are the results of an inductively coupled plasma-atomic emission spectroscopy (ICP-AES) analysis (*n *= 6) of wild-type cells grown in yeast extract-peptone-dextrose (YPD) medium with or without the metal supplements. Normalized accumulation is reported in parts per million (ppm) and *z *scores are reported for each element. Click here for file

## Figures and Tables

**Table 1:** Genes identified involved in vacuolar function

Gene	Function^a^
*CUP5*	Vacuolar H^+^-ATPase subunit
*TFP1*	Vacuolar H^+^-ATPase subunit
*TFP3*	Vacuolar H^+^-ATPase subunit
*VMA5*	Vacuolar H^+^-ATPase subunit
*VMA7*	Vacuolar H^+^-ATPase subunit
*VMA8*	Vacuolar H^+^-ATPase subunit
*VMA21*	Vacuolar H^+^-ATPase assembly
*VAM10*	Vacuole fusion (B)
*VPS41*	Golgi-to-vacuole vesicular transport (B)
*VPS16*	Golgi-to-vacuole vesicular transport (C)
*VPS33*	Golgi-to-vacuole vesicular transport (C)
*VPS9*	Golgi-to-vacuole vesicular transport (D)
*PEP12*	Golgi-to-vacuole vesicular transport (D)
*VPS45*	Golgi-to-vacuole vesicular transport (D)
*PEP7*	Golgi-to-vacuole vesicular transport (D)
*SNF8*	Vacuolar protein targeting (E)
*BRO1*	Vacuolar protein targeting (E)
*VPS36*	Vacuolar protein targeting (E)
*VPS4*	Endosome-to-vacuole vesicular transport (E)
*VAC14*	Vacuolar protein targeting
*VAM3*	Golgi-to-vacuole vesicular transport
*VPS53*	Endosome-to-Golgi vesicular transport
*VPS63*	Vacuolar protein targeting
*VPS64*	Vacuolar protein targeting
*VPS65*	Vacuolar protein targeting
*VPS66*	Vacuolar protein targeting
*AVT5*	Potential Vacuolar amino acid transporter

^a^The letter in parentheses indicates the assigned class of vacuolar biogenesis defect to which each strain belongs, if known.

**Table 2:** Genes identified involved in mitochondrial function

Gene	Function
*BCS1*	Cytochrome *bc*_(1) _complex biogenesis
*PDB1*	Pyruvate dehydrogenase activity
*DIA4*	Serine-tRNA ligase activity
*MTF1*	Mitochondrial RNA polymerase specificity factor
*PPA2*	Inorganic phosphatase
*MRPL35*	Mitochondrial ribosome subunit
*PET117*	Cytochrome oxidase assembly
*MRPL20*	Mitochondrial ribosome subunit
*NUC1*	DNA/RNA nuclease
*PTH1*	Peptidyl-tRNA hydrolase
*QCR2*	Ubiquinol cytochrome *c *reductase subunit
*MRP17*	Mitochondrial ribosome subunit
*RRF1*	Mitochondrial ribosome recycling factor
*RSM7*	Mitochondrial ribosome subunit
*COX10*	Heme *a *biosynthesis
*CBP3*	Ubiquinol cytochrome *c *reductase assembly
*CBP2*	Mitochondrial RNA splicing
*MSM1*	Methionyl-tRNA synthetase
*ATP10*	ATP synthase assembly
*CBP1*	Mitochondrial RNA processing
*MDL2*	ABC transporter
*YTA12*	Protein turnover
*IMP1*	Inner membrane protease subunit
*CYT2*	Cytochrome *c*_1 _heme lyase
*CBP4*	Ubiquinol-cytochrome *c *reductase assembly
*COQ5*	Ubiquinone biosynthesis
*COQ4*	Ubiquinone biosynthesis
*CTP1*	Inner membrane citrate transporter
*MRP13*	Mitochondrial ribosome subunit
*FIS1*	Mitochondrial division

**Table 3:** Final concentration of elements in growth medium

Element	Form added	Supplemented concentration	Final concentration
Calcium	CaCl_2_	1 mM	1.2 mM
Cobalt	CoCl_2_	5 μM	5.1 μM
Copper	CuCl_2_	100 μM	110 μM
Iron	-	-	19 μM
Magnesium	-	-	490 μM
Manganese	MnCl_2_	10 μM	11 μM
Nickel	NiCl_2_	125 μM	160 μM
Potassium	-	-	12 mM
Phosphorus	-	-	5.7 mM
Selenium	Na_2_SeO_3_	75 μM	75 μM
Sodium	-	-	16 mM
Sulfur	-	-	5.4 mM
Zinc	ZnCl_2_	100 μM	160 μM

Calcium, cobalt, copper, manganese, nickel, selenium and zinc were added to rich YPD medium (1% yeast extract, 2% peptone, 2% glucose) to facilitate their detection in cells by inductively coupled plasma-atomic emission spectroscopy (ICP-AES). These supplemented levels did not exceed 10% of the minimal growth inhibitory concentration determined for this strain (data not shown). The final concentration of these elements in the growth medium measured by ICP-AES is also shown and represents the supplemented levels plus those present in the YPD medium alone. The same lots of all medium components (such as yeast extract, peptone) were used throughout this study to maintain consistent growth conditions. Boron was added as H_2_BO_3 _at 181 μM and molybdenum was added as NaMoO_4 _at 10 μM. Despite this supplementation, levels of these two minerals accumulated by cells remained below the level of detection. -, not supplemented.
